# A medico-legal review of cases involving quadriplegia following cervical spine surgery: Is there an argument for a no-fault compensation system?

**DOI:** 10.4103/2152-7806.62261

**Published:** 2010-04-07

**Authors:** Nancy E. Epstein

**Affiliations:** Clinical Professor of Neurological Surgery, The Albert Einstein College of Medicine, Bronx, NY, USA and Chief of Neurosurgical Spine and Education, Winthrop University Hospital, Mineola, NY, USA

**Keywords:** Cervical spine surgery, Quadriplegia, Medico-legal liability suits, No-fault system

## Abstract

**Background:**

To determine whether patients who become quadriplegic following cervical spine surgery are adequately compensated by our present medico-legal system. The outcomes of malpractice suits obtained from *Verdict Search* (East Islip, NY, USA), a medico-legal journal, were evaluated over a 20-year period. Although the present malpractice system generously rewards many quadriplegic patients with substantial settlements/ Plaintiffs' verdicts, a subset receive lesser reimbursements (verdicts/settlements], while others with defense verdicts receive no compensatory damages.

**Methods:**

Utilizing *Verdict Search*, 54 cases involving quadriplegia following cervical spine surgery were reviewed for a 20-year interval (1988–2008). The reason(s) for the suit, the defendants, the legal outcome, and the time to outcome were identified. Operations included 25 anterior cervical procedures, 22 posterior cervical operations, 1 circumferential cervical procedure, and 6 cases in which the cervical operations were not defined.

**Results:**

The four most prominent legal allegations for suits included negligent surgery (47 cases), lack of informed consent (23 cases), failure to diagnose/treat (33 cases), and failure to brace (15 cases). Forty-four of the 54 suits included spine surgeons. There were 19 Plaintiffs' verdicts (average US $5.9 million, range US $540,000-US $18.4 million), and 20 settlements (average US $2.8 million, range US $66,500-US $12.0 million). Fifteen quadriplegic patients with defense verdicts received no compensatory damages. The average time to verdicts/settlements was 4.3 years.

**Conclusions:**

For 54 patients who were quadriplegic following cervical spine surgery, 15 (28%) with defense verdicts received no compensatory damages. Under a No-Fault system, quadriplegic patients would qualify for a “reasonable” level of compensation over a “shorter” time frame.

## INTRODUCTION

How can one determine whether the present medico-legal system adequately serves patients with negative outcomes following cervical spine surgery? In order to address this issue, we turned to a medico-legal journal, *Verdict Search* (East Islip, NY, USA), and identified 54 suits involving quadriplegia following cervical spine surgery over a 20-year interval (1988–2008). Quadriplegia was chosen as typically such outcomes result from adverse events. We asked: “Does the present medico-legal system adequately reimburse patients who are quadriplegic following cervical spine surgery?”

## METHODS

*Verdict Search:* Utilizing *Verdict Search*, 54 medico-legal suits involving quadriplegia following cervical spine surgery were identified over a 20-year interval (1988–2008). *Verdict Search* (East Islip, New York, USA), a medico-legal journal, reviews verdicts and/or settlements resulting from criminal and civil actions (excluding family case law); the latter includes personal injury, motor vehicle accidents, “slip and falls,” product liability, and medico-legal cases. A panel of lawyers and paralegals reviews medico-legal suits solicited by, or submitted to, *Verdict Search*. Cases are summarized in consultation with both Plaintiffs' and defendants' attorneys. *Verdict Search* reports 60%-80% of all available medico-legal suits from 6 states (New York, New Jersey, Pennsylvania, Florida, Texas, and California), while only 5%-10% of suits from other states are reviewed. However, *Verdict Search* reports on 60% of suits from the selected 6 states that result in Plaintiffs’ verdicts/settlements for more than US $1.0 million, and 100% over US $18.0 million.

Clinical Data: The 54 patients in this series included 36 men and 18 women averaging 46.4 years of age (range 5-82 years). Of these, 22 patients sustained preoperative trauma; 10 were involved in motor vehicle accidents, 2 sustained injuries (chiropractor and Murphy bed mishap), 1 involved an altercation, and 10 sustained falls. Surgical procedures included 25 anterior operations: 1-level anterior diskectomy/fusion (ADF) in 18 patients, 2-level ADF in 2 patients, 3-4-level ADF in 3 patients, 1-level anterior corpectomy/fusion (ACF) in 2 patients, and 22 posterior procedures (16 laminectomies/6 fusions). Additionally, one patient underwent an initial circumferential procedure [C6-C7], while 6 six cases did not specify the type of operation.

All 54 patients in this series exhibited complete quadriplegic deficits. Fourteen of these patients were part of an earlier review of cervical spine suits.

Location: The majority of suits originated in California (18 suits), New York (14 suits), Florida (4 suits), and Texas (3 suits). The remaining 15 suits were distributed amongst other states; 2 suits each in New Jersey, North Carolina, and Maine, and 1 per state for 9 other states.

## RESULTS

Who was sued? Spine surgeons were involved in 44 suits: alone or with a spine surgery group (15 suits), with hospitals (13 suits), with hospitals and other physicians/personnel (12 suits), with other physicians (3 suits), or with residents/chiropractors (1 suit). Spine surgeons were not included in 10 suits; these involved other physicians (1 suit), other physicians and hospitals (8 suits), and one bus company (1 suit).

### Allegations leading to suits

The four most prominent allegations leading to suits included negligent surgery (47 cases), failure to diagnose/treat (33 cases), lack of informed consent (23 cases), and failure to brace (15 cases). Other allegations included failure to perform postoperative studies (six cases), failure to place in a monitored unit postoperatively (three cases), anesthesia complications (three cases), failure to perform intraoperative somatosensory evoked potential monitoring [SSEP] (two cases), failure to treat intraoperative SSEP changes (one case), unnecessary surgery (two cases), use of non-FDA approved device (2 cases), wrong level surgery (one case), failure in credentialing (two cases), cerebrospinal fluid fistula/durotomy intraoperatively and failure to recognize ossification of the posterior longitudinal ligament (two cases), failure to recognize abscess (two cases), carotid laceration (one case), and penetration of cerebellum/brain damage (one case).

### Time to verdicts/settlements

The average time to verdicts/settlements was 4.3 years. The average times from filing a suit to settlement (2.8 years; range 1-8.4 years), and from filing to Plaintiffs’ verdicts (3.2 years; range 1-11.4 years) were shorter than the average 5.6-year interval (range 2-11.8 years) required to attain defense verdicts [Table 1]. The average number of trial days was similar whether Plaintiffs' or defense verdicts were rendered (12.3-12.7 days), but the average jury deliberation times were longer for Plaintiffs' verdicts (31.25 hours) versus defense verdicts (14 hours).

**Table 1:** Times to verdict or settlement

Outcomes	Average (years)	Range (years)
Settlements	2.8	1-8.4
Plaintiffs’ verdicts	3.2	1-11.4
Defense verdicts	5.6	2-11.8

### Reimbursement for Plaintiffs' verdicts and settlements

The level of reimbursement for quadriplegic patients following Plaintiffs' verdicts and settlements varied markedly [Table 2]. For the 19 Plaintiffs' verdicts, the average reimbursement was US $5.9 million (range US $540,000-US $18.4 million), but 6 patients received under less than US $1.7 million. Settlements for 20 patients averaged US $2.8 million (range US $66,500-US $12.0 million), but 10 patients received under US $1.7 million. Most importantly, the 15 patients with defense verdicts received no compensation whatsoever.

**Table 2:** Verdicts/settlements: Payments and non-payments

Outcome	#No.	Average (millions)	Range (millions)	< 1.7 (million)
Plaintiffs’ verdicts	19	US $5.9	US $540 K-US $18.4	6
Settlements	20	US $2.8	US $66.5K-US $12.0	10
Defense verdicts	15	US $0.00	US $0.00	15

Example cases: To illustrate the vagaries of the current medico-legal system, consider the following 3 cases.

Case 1: A 67-year-old male with cervical spondylosis and neck pain only, who underwent a cervical laminectomy, became quadriplegic immediately postoperatively. A defense verdict was rendered, and he received nothing.

Case 2: A 42-year-old male with progressive myelopathy, underwent a C4 anterior corpectomy/fusion [C3-C5 strut grafting]; he was quadriplegic immediately postoperatively. He sued for US $100,000 in prior costs and an anticipated US $1.7 million in future costs. Again, a defense verdict was rendered, and the patient received no compensation.

Case 3: An 82-year-old female with mild myeloradiculopathy underwent a routine cervical laminectomy (without fusion) for stenosis; she was quadriplegic immediately postoperatively. In this case, which bears marked similarities to the prior two cases, the patient received a Plaintiffs' verdict (US $1.394 million).

## DISCUSSION

One major disadvantage of the present “tort” malpractice system is its failure to compensate a significant group of truly injured patients. For the 54 quadriplegic patients in this series, all 15 (28%) with defense verdicts received no reimbursement. In addition, 6 of 19 with Plaintiffs' verdicts, and 10 of 20 with settlements, received less than US $1.7 million in compensatory damages.

A second disadvantage of our current system is the extended time required for verdicts/settlements to be rendered. In this series, Plaintiffs' verdicts (average 3.2 years) and settlements (average 2.8 years) were rendered in substantially less time than defense verdicts (5.6 years); however, for all 3 groups, ranges were remarkably long high (up to 8.4-11.8 years). Under a No-Fault system, patients who experience significant injuries could be covered under “Accelerated Compensable Events (ACE)”, defined as those events that are normally avoidable given an adequate standard of care (e.g., quadriplegia, wrong-sided surgery, wrong-level surgery, death, etc.).[1,2] ACE could be identified and reasonably compensated within a shorter period of time.[1–4] For example, Swedish patients apply for No-Fault system claims utilizing forms provided in clinics and hospitals where their physicians/health professionals help fill them out.[5] A claim is reviewed by 1 or more specialists and a decision rendered, usually within 6 months. Those compensated, about 40% of claims, are reimbursed in a uniform manner utilizing a fixed benefit schedule (economic/non economic damages).[5]

A third major disadvantage of the present system includes large payouts to lawyers and other third parties, rather than to the injured Plaintiffs. Siebst, Friedman, and Singh[6] reported that Plaintiffs' received US $116 million, while additional defense attorney fees were US $24.0 million, and expert fees were US $3.4 million.[6,7] Alternatively, No-Fault systems like that in New Zealand are less expensive as they remove legal barriers to filing/adjudicating claims.[8] Further, although frivolous suits may be more commonplace under a No-Fault system, they are typically less costly, and therefore, would not increase medical costs.[5,8–10]

Finally, the fourth major disadvantage of the present “tort” malpractice system is the failure to link malpractice with a reduction in adverse events; defensive medicine is still being practiced while medical errors remain underestimated/undetected.[1,3–9,11,12] Morris, Carrillo, Jenkins, et al reported that 130 patients experienced 390 adverse events, or an average of three system failures per patient.[1] Five adverse events accounted for 75% of failures: patient management (104 failures), communication (89 failures), administration (33 failures), documentation (32 failures), and behavior (23 failures). A No-Fault system would probably lower the barriers toward disclosing medical errors, and would also foster greater cooperation in reducing future errors.

## CONCLUSION

In the cases reviewed, 15 of 54 patients (28%) who became quadriplegic following cervical spine surgery were not compensated by the present medico-legal system. This is a clear example of how the current system does not serve the patients' best interests. A No-Fault system would allow all truly injured patients to be reasonably compensated within a shorter time frame, while maximal efforts could be geared toward reducing/avoiding future adverse events.
